# GLP-1 Receptor Agonists and Sight-Threatening Ophthalmic Complications in Patients With Type 2 Diabetes

**DOI:** 10.1001/jamanetworkopen.2025.26321

**Published:** 2025-08-11

**Authors:** David J. Ramsey, Bhargav Makwana, Sourbha S. Dani, Manav Patel, Krisha Panchal, Jui Shah, Sumanth Khadke, Ashish Kumar, Tirth Patel, Mikhail N. Kosiborod, Gregg C. Fonarow, Kathryn Moynihan Ramsey, Anju Nohria, Javed Butler, Sarju Ganatra

**Affiliations:** 1 Division of Ophthalmology, Department of Surgery, UMass Chan–Lahey School of Medicine, Burlington, Massachusetts; 2 Department of Ophthalmology, Tufts University School of Medicine, Boston, Massachusetts; 3 Department of Biomedical Sciences and Disease, New England College of Optometry, Boston, Massachusetts; 4 Department of Medicine, Lahey Hospital & Medical Center, Beth Israel Lahey Health, Burlington, Massachusetts; 5 Division of Cardiovascular Medicine, Department of Medicine, Lahey Hospital & Medical Center, Beth Israel Lahey Health, Burlington, Massachusetts; 6 Department of Cardiovascular Medicine, Mayo Clinic, Rochester, Minnesota; 7 Department of Cardiovascular Medicine, Saint Luke’s Mid America Heart Institute and the University of Missouri, Kansas City; 8 Division of Cardiology, Department of Medicine, Ronald Reagan–UCLA (University of California, Los Angeles) Medical Center, Los Angeles; 9 Department of Medicine, Division of Endocrinology, Metabolism and Molecular Medicine, Northwestern University Feinberg School of Medicine, Chicago, Illinois; 10 Brigham and Women’s Hospital Heart and Vascular Center, Harvard Medical School, Boston, Massachusetts; 11 Department of Cardiovascular Medicine, Baylor Scott and White Research Institute, Dallas, Texas; 12 Department of Cardiovascular Medicine, University of Mississippi, Jackson

## Abstract

**Importance:**

Glucagon-like peptide-1 receptor agonists (GLP-1 RAs) are associated with increased risk of diabetic retinopathy (DR) and nonarteritic anterior ischemic optic neuropathy (NAION). The risk of sight-threatening complications associated with GLP-1 RAs is underexamined.

**Objective:**

To investigate whether the use of GLP-1 RAs in patients with T2D is associated with the development of DR, NAION, or DR complications.

**Design, Setting, and Participants:**

This retrospective cohort study of adults (aged ≥18 years) with T2D and a recent hemoglobin A_1c_ level of 6.5% or higher was conducted between January 1, 2015, and September 30, 2022, using the TriNetX database. The cohort was divided into 2 groups, adjusted for baseline characteristics through propensity score matching (PSM), based on whether the individuals received prescriptions for a GLP-1 RA. The statistical analysis was conducted on October 10, 2024.

**Exposures:**

At least 2 prescriptions of a GLP-1 RA given 6 months apart.

**Main Outcomes and Measures:**

Cox proportional hazard regression models were used to evaluate the primary outcome: association between GLP-1 RAs and the risk of incident DR, NAION, or sight-threatening complications over a 2-year follow-up period.

**Results:**

After PSM, 185 066 individuals (mean [SD] age, 59.0 [12.5] years; 93 389 females [50.5%]) were prescribed GLP-1 receptor agonists. Use of GLP-1 RAs was associated with an increased incidence of DR (hazard ratio [HR], 1.07; 95% CI, 1.03-1.11), while no statistically significant difference was observed in the risk of NAION (HR, 1.26; 95% CI, 0.94-1.70). In a subgroup analysis of 32 695 patients with preexisting DR, GLP-1 RAs were not associated with progression to proliferative DR (HR, 1.06; 95% CI, 0.97-1.15) or diabetic macular edema (HR, 0.98; 95% CI, 0.95-1.01) but were associated with a lower occurrence of vitreous hemorrhages (HR, 0.74; 95% CI, 0.68-0.80), neovascular glaucoma (HR, 0.78; 95% CI, 0.68-0.88), or blindness (HR, 0.77; 95% CI, 0.73-0.82).

**Conclusions and Relevance:**

In this cohort study of individuals with T2D, GLP-1 RA use was associated with a modestly increased risk of incident DR; however, fewer patients experienced sight-threatening DR complications, including blindness, even among those with preexisting DR. These findings suggest that all patients with T2D treated with GLP-1 RAs, regardless of preexisting DR, should be regularly screened and monitored for potential complications of T2D.

## Key Points

**Question:**

Is the use of glucagon-like peptide-1 receptor agonists (GLP-1 RAs) in patients with type 2 diabetes (T2D) associated with an increased risk of sight-threatening diabetic retinopathy (DR) or nonarteritic anterior ischemic optic neuropathy (NAION)?

**Findings:**

This cohort study of 185 066 individuals prescribed treatment with GLP-1 RAs revealed an association with slightly higher risk of developing incident DR but a similar incidence of NAION and fewer serious DR-associated complications, including new-onset blindness.

**Meaning:**

These findings suggest that all patients with T2D treated with GLP-1 RAs, regardless of preexisting DR, should receive regular screening and monitoring for potential complications of T2D.

## Teaser

This cohort study examines the occurrence of diabetic retinopathy, ischemic optic nerve disease, or other ocular complications within 2 years of glucagon-like peptide-1 receptor agonist (GLP-1 RA) use among patients with type 2 diabetes.

## Introduction

Glucagon-like peptide-1 receptor agonists (GLP-1 RAs) are an important class of antihyperglycemic medications approved by the US Food and Drug Administration for the treatment of type 2 diabetes (T2D). They have been highly effective in helping patients with T2D meet glycemic targets,^1^ achieve substantial weight loss,^2^ and improve cardiometabolic and kidney outcomes in high-risk subgroups.^3,4,5,6,7^ As a result, the American Diabetes Association recommends GLP-1 RAs as 1 of 2 possible first-line therapies for patients with T2D who have, or are at high risk for, atherosclerotic cardiovascular disease.^8^

Despite the effectiveness of GLP-1 RAs in reducing glycemic levels and improving hemoglobin A_1c_ (HbA_1c_) levels, some concerning adverse effects have been reported.^1,2,3,4^ Randomized clinical trials and observational studies have linked GLP-1 RAs with the development of diabetic retinopathy (DR)^5,9^ and an increased risk of nonarteritic anterior ischemic optic neuropathy (NAION) within the first year of use.^10^ However, a recent meta-analysis of randomized clinical trials suggests that GLP-1 RAs may not increase the risk of DR.^11^ Furthermore, a large cohort study of patients with T2D, with and without obesity, did not find an increased risk of NAION associated with these agents.^12^ A major limitation of prior studies of GLP-1 RAs in patients with T2D is that they did not specifically assess for sight-threatening complications from DR, which account for most of the long-term ocular morbidity associated with T2D. This retrospective cohort study aimed to investigate whether GLP-1 RA use is associated with the development of DR, NAION, or DR complications in patients with T2D.

## Methods

The Lahey Hospital and Medical Center Institutional Review Board deemed this cohort study exempt from ethics review and the informed consent requirement since the analysis used aggregate, anonymized data from a research network database. The study findings are reported in accordance with Strengthening the Reporting of Observational Studies in Epidemiology (STROBE) reporting guideline.

### Data Source

We used the TriNetX database, a multicenter federated health research network that aggregates anonymized data from electronic health records (EHRs) drawn from more than 120 participating health care organizations, including academic medical centers, specialty physician practices, and community hospitals, covering approximately 275 million patients. While the data are in aggregate and deidentified, the built-in analytics of the database enable cohort selection and matching, analysis of the incidence and prevalence of events within a cohort, and comparison of characteristics and outcomes between matched cohorts. More information can be found on the TriNetX website.^13,14^

Race and ethnicity were self-identified in the EHRs. The categories we used in matching cohorts were Asian, Black or African American, Hispanic or Latino, not Hispanic or Latino, White, and unknown. Race and ethnicity data were collected because of their potential implications for the rate of GLP-1 RA prescriptions or study outcomes.

### Population and Design

Between January 1, 2015, and September 30, 2022, we conducted a retrospective cohort study of patients aged 18 years or older with T2D and a recent HbA_1c_ of 6.5% or higher identified from the TriNetX database (to convert HbA_1c_ to proportion of total hemoglobin, multiply by 0.01). Patients were sorted into 2 cohorts based on whether they had received a prescription for a GLP-1 RA (semaglutide, lixisenatide, tirzepatide, dulaglutide, liraglutide, or exenatide): those with GLP-1 RA prescriptions were considered the treatment group, whereas those without GLP-1 RA prescriptions were considered the control group. We included only patients with at least 2 GLP-1 RA prescriptions given at least 6 months apart. The index event was defined as the date of the second GLP-1 RA prescription for the treatment group and as the date of meeting the inclusion criteria for the control group. Individuals with more than 2 prescriptions were included in the treatment group based on the query criteria. Those with only 1 prescription or 2 prescriptions given less than 6 months apart were excluded. Cohorts were matched using propensity scores for clinically relevant variables (eAppendix and eTable 1 in Supplement 1). Outcomes were assessed within a 2-year window after the index event.

### End Points

The primary end points were the association between GLP-1 RAs and the risk of incident DR, NAION, or sight-threatening complications over a 2-year follow-up period. The study quantified the incidence of diabetic eye disease and other ischemic retinal vascular disorders, including incident DR, neovascular glaucoma, and NAION, by using *International Statistical Classification of Diseases and Related Health Problems, Tenth Revision (ICD-10)* or *Current Procedural Terminology* codes in the 2 cohorts (eTable 2 in Supplement 1). A subgroup analysis was conducted on individuals with a prior diagnosis of DR to assess the risk of disease progression to proliferative diabetic retinopathy (PDR), the development of a vitreous hemorrhage, or new-onset diabetic macular edema (DME). We also assessed whether patients with preexisting DR received treatment with agents targeting vascular endothelial growth factor (VEGF) or, more broadly, with intravitreal injections, laser panretinal photocoagulation, or retinal surgery via vitrectomy either individually or as a composite treatment outcome. Additionally, we examined the incidence of new-onset blindness, including cases coded as low vision or legal blindness, in both the entire cohort and the subset of patients with prior DR.

### Statistical Analysis

Continuous variables are presented as mean (SD), and categorical variables are presented as number (%). One-to-one propensity score matching (PSM) was performed using greedy nearest-neighbor matching with a caliper of 0.1 times the pooled SD of the linear propensity scores to control for baseline differences between the study groups. The standard mean difference is a quantitative method used to represent the difference between the means of 2 groups in terms of SD units; it assesses the balance in measured variables in a sample weighted by the inverse probability of treatment. The variables were chosen according to their potential association with the study outcomes selected.

After PSM, outcomes were compared between the 2 cohorts using absolute and relative risk difference. Kaplan-Meier curves and Cox proportional hazards regression models were used for survival analysis. Statistical significance was set at a 2-sided *P* < .05. Statistical analyses were performed on October 10, 2024, using an integrated R, version 4.0.3 (R Project for Statistical Computing) on the TriNetX platform.

To strengthen the reliability of the observational data, we evaluated falsification outcomes, such as the incidence of appendicitis, within the same 2-year follow-up time frame for the GLP-1 RA and comparator groups. We also calculated E-values for primary and secondary outcomes to assess potential confounding from unmeasured factors. Higher E-values indicate that unmeasured confounders with a greater influence on the outcome of interest would be required to negate the observed association between exposure and outcome.

Recognizing that a longer duration of known diabetes was associated with an increased risk of incident DR, we accounted for the duration of T2D by comparing the incidence of diabetic eye disease in individuals with GLP-1 RA prescriptions who had a diagnosis of T2D within less than 10 years vs greater than 10 years after the index event.^15^ A duration of 10 years was selected because previous studies have shown a direct association between HbA_1c_ levels and 10-year incidence of DR.^16^ To ensure that the severity of DR did not confound the study results for the preexisting DR subgroup, we did a look-back on individuals with vs without GLP-1 RA prescriptions to assess the number of individuals with varying severity of DR at baseline.

## Results

### Baseline Characteristics

After PSM, 185 066 individuals were prescribed GLP-1 RAs. These patients had a mean (SD) age of 59.0 (12.5) years and included 93 389 females (50.5%) and 91 677 males (49.5%), with 6070 individuals who identified as Asian (3.3%), 33 306 as Black or African American (18.0%), 17 891 as Hispanic or Latino (9.7%), 128 515 as not Hispanic or Latino (69.4%), and 113 256 as White (61.2%) (Table 1). The matched control group of individuals without GLP-1 RA prescriptions had a mean (SD) age of 59.3 (13.9) years and included 93 757 females (50.7%) and 91 309 males (49.3%), with 5716 individuals who identified as Asian (3.1%), 33 209 as Black or African American (17.9%), 17 693 as Hispanic (9.6%), 128 769 as not Hispanic or Latino (69.6%), and as 114 315 White (61.8%). The *ICD-10* and Veterans Affairs health care–specific codes for these baseline characteristics are presented in eTable 1 in Supplement 1.

**Table 1.** Baseline Characteristics of Patients With Type 2 Diabetes Before and After Propensity Score Matching

Characteristic	Before PSM			After PSM		
	With GLP-1 RA prescriptions, No. (%) (n = 280 593)	Without GLP-1 RA prescriptions, No. (%) (n = 1 399 684)	Standardized difference	With GLP-1 RA prescriptions, No. (%) (n = 185 066)	Without GLP-1 RA prescriptions, No. (%) (n = 185 066)	Standardized difference
Demographics						
Age, mean (SD), y	58.0 (12.3)	61.9 (15.1)	0.277	59.0 (12.5)	59.3 (13.9)	0.028
Sex						
Female	145 019 (51.7)	613 628 (43.8)	0.157	93 389 (50.5)	93 757 (50.7)	0.004
Male	135 574 (48.3)	786 056 (56.2)	0.139	91 677 (49.5)	91 309 (49.3)	0.008
Race and ethnicity^a^						
Asian	8088 (2.9)	83 142 (5.9)	0.149	6070 (3.3)	5716 (3.1)	0.011
Black or African American	50 182 (17.9)	231 167 (16.5)	0.036	33 306 (18.0)	33 209 (17.9)	0.001
Hispanic or Latino	27 154 (9.7)	139 875 (10.0)	0.011	17 891 (9.7)	17 693 (9.6)	0.004
Not Hispanic or Latino	197 797 (70.5)	922 855 (65.9)	0.098	128 515 (69.4)	128 769 (69.6)	0.003
White	174 381 (62.1)	787 430 (56.3)	0.120	113 256 (61.2)	114 315 (61.8)	0.012
Comorbidities						
Dyslipidemia	240 406 (85.7)	687 772 (49.1)	0.847	151 500 (81.9)	153 450 (82.9)	0.028
Hypertension	236 233 (84.2)	822 105 (58.7)	0.588	151 086 (81.6)	153 047 (82.7)	0.028
Ischemic heart diseases	70 524 (25.1)	299 960 (21.4)	0.088	46 973 (25.4)	47 746 (25.8)	0.010
Atrial fibrillation or flutter	23 156 (8.3)	125 265 (8.9)	0.025	16 313 (8.8)	16 582 (9.0)	0.005
Stroke	14 784 (5.3)	73 803 (5.3)	0.001	10 337 (5.6)	10 384 (5.6)	0.001
Peripheral vascular diseases	8501 (3.0)	29 879 (2.1)	0.056	5735 (3.1)	5972 (3.2)	0.007
CKD	55 081 (19.6)	199 192 (14.2)	0.144	35 718 (19.3)	36 617 (19.8)	0.012
COPD	82 728 (29.5)	230 628 (16.5)	0.313	50 525 (27.3)	50 917 (27.5)	0.005
Benign neoplasms	84 306 (30.0)	167 466 (12.0)	0.455	48 855 (26.4)	48 782 (26.4)	0.001
Malignant neoplasms	109 702 (39.1)	310 278 (22.2)	0.374	66 903 (36.2)	66 823 (36.1)	0.001
Personal history of nicotine dependence	46 852 (16.7)	125 620 (9.0)	0.232	26 969 (14.6)	26 916 (14.5)	0.001
Tobacco use	18 287 (6.5)	38 178 (2.7)	0.181	9711 (5.2)	9543 (5.2)	0.004
Medication use						
Insulin	178 341 (63.6)	501 520 (35.8)	0.577	108 236 (58.5)	109 517 (59.2)	0.014
Metformin	234 768 (83.7)	444 171 (31.7)	1.236	143 999 (77.8)	146 838 (79.3)	0.037
Glipizide	73 495 (26.2)	121 824 (8.7)	0.474	40 933 (22.1)	41 613 (22.5)	0.009
Sitagliptin	65 279 (23.3)	84 857 (6.1)	0.501	32 739 (17.7)	32 426 (17.5)	0.004
Empagliflozin	60 058 (21.4)	20 251 (1.4)	0.661	14 558 (7.9)	13 168 (7.1)	0.029
Canagliflozin	25 510 (9.1)	9942 (0.7)	0.396	6808 (3.7)	6117 (3.3)	0.020
Glyburide	19 498 (6.9)	41 677 (3.0)	0.184	12 361 (6.7)	12 817 (6.9)	0.010
Dapagliflozin	25 635 (9.1)	11 995 (0.9)	0.387	7311 (4.0)	6673 (3.6)	0.018
Tirzepatide	4387 (1.6)	0	0.178	359 (0.2)	0	0.062
Antilipemic agents	231 637 (82.6)	597 510 (42.7)	0.904	145 314 (78.5)	147 711 (79.8)	0.032
Loop diuretics	64 241 (22.9)	222 373 (15.9)	0.178	42 316 (22.9)	43 257 (23.4)	0.012
Spironolactone	24 483 (8.7)	54 878 (3.9)	0.198	14 223 (7.7)	14 156 (7.6)	0.001
ACE inhibitors	149 701 (53.4)	380 045 (27.2)	0.554	93 915 (50.7)	96 051 (51.9)	0.023
Angiotensin II inhibitors	97 349 (34.7)	226 471 (16.2)	0.435	58 249 (31.5)	58 529 (31.6)	0.003
Sacubitril	3859 (1.4)	6857 (0.5)	0.092	1868 (1.0)	1797 (1.0)	0.004
Antineoplastics	24 701 (8.8)	64 302 (4.6)	0.169	14 966 (8.1)	14 984 (8.1)	0.001
Laboratory values						
LDL cholesterol, mean (SD), mg/dL	85.6 (36.7)	94.0 (39.2)	0.221	85.7 (36.4)	93.2 (39.7)	0.198
BNP ≥150 pg/mL	37 469 (13.4)	138 826 (9.9)	0.045	23 307 (12.6)	26 144 (14.1)	0.045
NT-proBNP ≥450 pg/mL	22 626 (8.1)	76 539 (5.5)	0.103	13 925 (7.5)	14 930 (8.1)	0.020
HbA_1c_ ≥6.5%	278 326 (99.2)	534 952 (38.2)	1.745	183 229 (99.0)	145 874 (78.8)	0.185
HbA_1c_, mean (SD), %	8.0 (1.9)	7.7 (2.0)	0.180	7.9 (1.9)	7.6 (1.9)	0.128
Iron, mean (SD), μg/dL	67.0 (35.4)	64.6 (41.7)	0.062	67.4 (35.4)	65.1 (39.1)	0.060
CRP, mean (SD), mg/L	23.5 (47.5)	37.7 (65.5)	0.248	23.7 (47.7)	29.4 (55.6)	0.109
LVEF, mean (SD), %	59.2 (12.1)	56.3 (14.7)	0.222	59.6 (11.7)	56.1 (14.7)	0.258
BMI ≥30	141 429 (50.4)	363 942 (26.0)	0.519	86 360 (46.7)	87 834 (47.5)	0.016

Abbreviations: ACE, angiotensin-converting enzyme; BMI, body mass index (calculated as weight in kilograms divided by height in meters squared); BNP, B-type natriuretic peptide; CKD, chronic kidney disease; COPD, chronic obstructive pulmonary disease; CRP, C-reactive protein; GLP-1 RA, glucagon-like peptide-1 receptor agonist; HbA_1c_, hemoglobin A_1c_; LDL, low-density lipoprotein; LVEF, left ventricular ejection fraction; NT-proBNP, N-terminal pro–brain natriuretic peptide; PSM, propensity score matching.

SI conversion factor: To convert BNP to nanogram per liter, multiply by 1.0; CRP to milligram per liter, multiply by 10; HbA_1c_ to proportion of total hemoglobin, multiply by 0.01; iron to micromole per liter, multiply by 0.179; LDL cholesterol to millimoles per liter, multiply by 0.0259.

^a^ Race and ethnicity were self-reported in the organizational electronic health records and obtained from TriNetX database.

Before PSM, individuals with GLP-1 RA prescriptions were younger; female; identified as not Hispanic or Latino or White; had higher rates of hypertension, dyslipidemia, and ischemic heart disease; and greater use of insulin, metformin, glipizide, sodium-glucose cotransporter 2 inhibitors, and renin-angiotensin-aldosterone system inhibitors compared with individuals without GLP-1 RA prescriptions. Following PSM, the 2 cohorts were well-matched for demographics, comorbidities, baseline medication use, and laboratory values, as indicated by a standardized difference of less than 0.1 for most baseline characteristics included in the matching process (Table 1; eMethods in Supplement 1).

### Incidence of DR and NAION

During the 2-year follow-up period, 5037 (2.7%) individuals in the GLP-1 RA group developed incident DR compared with 4938 (2.7%) individuals in the group without GLP-1 RA prescriptions (n = 185 066 per group; HR, 1.07; 95% CI, 1.03-1.11; *P* = .001) (Table 2). The risk of NAION was modestly higher among the treatment group than the control group (96 [0.1%] vs 79 [<0.1%] events; HR, 1.26 [95% CI, 0.94-1.70]; *P* = .12), as was the risk of a broader set of ischemic optic nerve conditions (243 [0.1%] vs 230 [0.1%] events; HR, 1.10 [95% CI, 0.92-1.32]; *P* = .31). However, these differences were not statistically significant, and the wide CIs suggested imprecision due to limited event counts. The incidence of all-cause blindness was less often coded for individuals with GLP-1 RA prescriptions compared with those without GLP-1 RA prescriptions (2313 [1.2%] vs 3051 [1.6%] events; HR, 0.77 [95% CI, 0.73-0.82]; *P* < .001), a 24.2% relative reduction in the risk of blindness.

**Table 2.** Comparison of Ocular Complications Among Patients With T2D With vs Without GLP-1 RA Prescriptions

Outcome^a^	T2D with GLP-1 RA prescriptions, No. (%) (n = 185 066)	T2D without GLP-1 RA prescriptions, No. (%) (n = 185 066)	RD (95% CI)	RRR, %	HR (95% CI)	*P* value	E-value for HR	E-value for lower CI of HR
DR	5037 (2.7)	4938 (2.7)	0.001 (−0.000 to 0.002)	−2.00	1.07 (1.03 to 1.11)	.001	1.35	1.21
NAION	96 (0.1)	79 (0.0)	0.000 (−0.000 to 0.000)	−21.50	1.26 (0.94 to 1.70)	.12	1.84	1.33
Neovascular glaucoma	446 (0.2)	559 (0.3)	−0.001 (−0.001 to −0.000)	20.21	0.82 (0.73 to 0.93)	.002	1.72	2.09
Blindness	2313 (1.2)	3051 (1.6)	−0.004 (−0.005 to −0.003)	24.19	0.77 (0.73 to 0.82)	<.001	1.91	2.07

Abbreviations: DR, diabetic retinopathy; GLP-1 RA, glucagon-like peptide-1 receptor agonist; HR, hazard ratio; NAION, nonarteritic anterior ischemic optic neuropathy; RD, risk difference; RRR, relative risk reduction; T2D, type 2 diabetes.

^a^ After propensity matching.

### Risk of DR Progression and Sight-Threatening Complications

We evaluated the risk of retinopathy progression, sight-threatening complications, and DR treatments in a subgroup of patients with T2D and preexisting DR at the time of GLP-1 RA prescription. Initially, 44 241 individuals with DR received GLP-1 RA prescriptions, while 124 760 individuals did not. After PSM, 32 695 individuals remained in each group and were well-matched for demographics, comorbidities, baseline medication use, and laboratory values (Table 3).

**Table 3.** Comparison of Ocular Complications and Other Outcomes Among Patients With Preexisting Diabetic Retinopathy With vs Without GLP-1 RA Prescriptions

Outcome^a^	Preexisting DR with GLP-1 RA prescriptions (n = 32 695), No. (%)	Preexisting DR without GLP-1 RA prescriptions (n = 32 695), No. (%)	RD (95% CI)	RRR, %	HR (95% CI)	*P* value	E-value for HR	E-value for lower CI of HR
PDR	1121 (3.4)	1039 (3.2)	0.002 (−0.001 to 0.005)	−7.89	1.06 (0.97 to 1.15)	.18	1.31	1.19
DME	7390 (22.6)	7425 (22.7)	−0.001 (−0.007 to 0.005)	0.47	0.98 (0.95 to 1.01)	.25	1.12	1.05
Neovascular glaucoma	434 (1.3)	562 (1.7)	−0.004 (−0.006 to −0.002)	22.78	0.78 (0.68 to 0.88)	<.001	1.90	2.28
NAION	34 (0.1)	38 (0.1)	−0.000 (−0.001 to 0.000)	10.53	0.91 (0.57 to 1.45)	.69	1.43	2.89
Vitreous hemorrhage	1135 (3.5)	1542 (4.7)	−0.012 (−0.015 to −0.009)	26.39	0.74 (0.68 to 0.80)	<.001	2.05	2.28
Panretinal laser photocoagulation	698 (2.1)	894 (2.7)	−0.006 (−0.008 to −0.004)	21.92	0.79 (0.71 to 0.87)	<.001	1.86	2.16
Vitrectomy	445 (1.4)	722 (2.2)	−0.008 (−0.010 to −0.006)	38.40	0.62 (0.55 to 0.70)	<.001	2.61	3.03
Treatment with anti-VEGF medications	296 (0.9)	355 (1.1)	−0.002 (−0.003 to −0.000)	16.62	0.84 (0.72 to 0.98)	.03	1.66	2.12
Composite treatment^b^	2341 (7.2)	2547 (7.8)	−0.006 (−0.010 to −0.002)	8.10	0.92 (0.87 to 0.97)	.003	1.40	1.57
Blindness	1280 (3.9)	1833 (5.6)	−0.016 (−0.020 to −0.013)	30.17	0.70 (0.65 to 0.75)	<.001	2.22	2.45

Abbreviations: DME, diabetic macular edema; DR, diabetic retinopathy; GLP-1 RA, glucagon-like peptide-1 receptor agonist; HR, hazard ratio; NAION, nonarteritic anterior ischemic optic neuropathy; PDR, proliferative diabetic retinopathy; RD, risk difference; RRR, relative risk reduction; VEGF, vascular endothelial growth factor.

^a^ After propensity matching.

^b^ Composite treatment consists of treatment of DR with anti-VEGF agents, panretinal laser photocoagulation, or vitrectomy.

Treatment with GLP-1 RAs was not associated with progression to PDR (1121 of 32 695 [3.4%] vs 1039 of 32 695 [3.2%] events; HR, 1.06 [95% CI, 0.97-1.15]; *P* = .18) or new-onset DME (7390 [22.6%] vs 7425 [22.7%] events; HR, 0.98 [95% CI, 0.95-1.01]; *P* = .25) (Table 3). There was also a reduced occurrence of vitreous hemorrhage (1135 [3.5%] vs 1542 [4.7%] events; HR, 0.74 [95% CI, 0.68-0.80]; *P* < .001) and neovascular glaucoma (434 [1.3%] vs 562 [1.7%] events; HR, 0.78 [95% CI, 0.68-0.88]; *P* < .001) as well as a reduced need for treatment with anti-VEGF inhibitors (296 [0.9%] vs 355 [1.1%] events; HR, 0.84 [95% CI, 0.72-0.98]; *P* = .03), panretinal laser photocoagulation (698 [2.1%] vs 894 [2.7%] events; HR, 0.79 [95% CI, 0.71-0.87]; *P* < .001), a vitreoretinal procedure for DR (445 [1.4%] vs 722 [2.2%] events; HR, 0.62 [95% CI, 0.55-0.70]; *P* < .001), or composite treatment involving any of these procedures (2341 [7.2%] vs 2547 [7.8%] events; HR, 0.92 [95% CI, 0.87-0.97]; *P* = .003).

In this subgroup (Figure), there was no difference in the rate of ischemic optic neuropathy between groups (34 [0.1%] vs 38 [0.1%] events; HR, 0.91 [95% CI, 0.57-1.45]; *P* = .69), even when more broadly defined (78 [0.2%] vs 96 [0.3%] events; HR, 0.82 [95% CI, 0.61-1.11]; *P* = .21). The incidence of all-cause blindness was even less often coded in those patients who had preexisting DR and received GLP-1 RA prescriptions compared with those without GLP-1 RA prescriptions (1280 [3.9%] vs 1833 [5.6%] events; HR, 0.70 [95% CI, 0.65-0.75], *P* < .001; *Z* = −2.166, *P* = .02), a 30.2% relative risk reduction in blindness.

**Figure.**
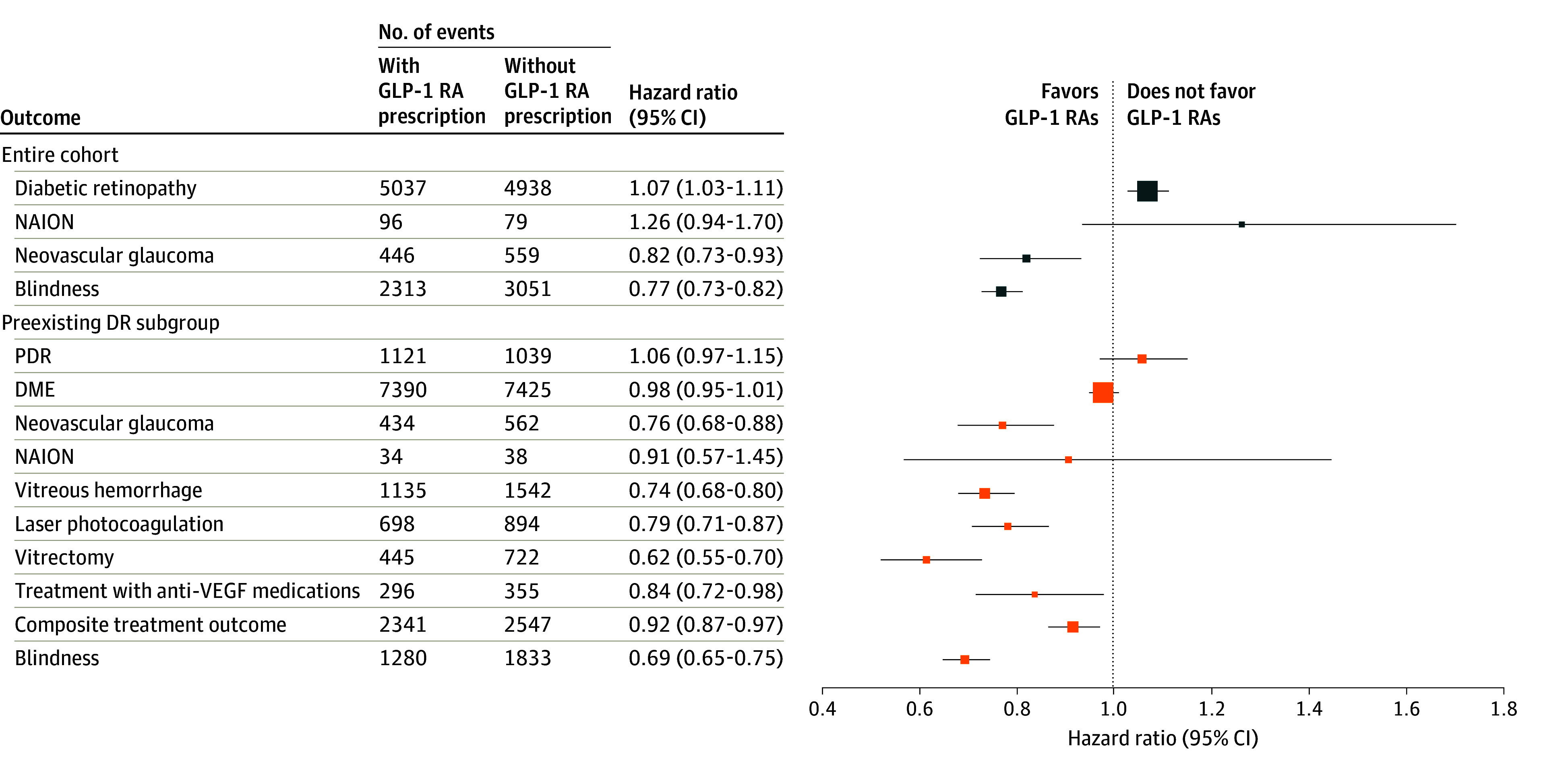
Ophthalmic End Points and Development of Sight-Threatening Complications in Patients With Glucagon-Like Peptide-1 Receptor Agonist (GLP-1 RA) Prescriptions Outcomes in the entire cohort (n = 185 066 individuals per group) are shown in blue, while outcomes for the subgroup with preexisting diabetic retinopathy (DR) (n = 32 695 individuals per group) are shown in orange. Marker size is proportional to the mean total number of events in each category. DME indicates diabetic macular edema; NAION, nonarteritic anterior ischemic optic neuropathy; PDR, proliferative diabetic retinopathy; and VEGF, vascular endothelial growth factor.

### Sensitivity Analyses

The falsification outcome of appendicitis during the follow-up period showed no difference between the treatment and control cohorts, regardless of preexisting DR (Table 4). By contrast, the higher E-values for the study outcomes, shown in Table 2 and Table 3, suggest that substantial unmeasured confounding would be required to fully explain the observed associations. In our additional analyses of ophthalmologic end points among the treatment group, both the well-matched cohorts (n = 68 424 per group) with a T2D diagnosis less than 10 years vs greater than 10 years after the index event showed similar incidence of diabetic eye disease over a 2-year follow-up (eg, DME: 5127 [7.5%] vs 5185 [7.6%] events; HR, 0.99 [95% CI, 0.95-1.03]; *P* = .59) (eTable 3 in Supplement 1), indicating that the role of the duration of diabetes in our study outcomes was relatively negligible. In the preexisting DR subgroup, the severity of diabetic retinopathy was similar between individuals with and without GLP-1 RA prescriptions at baseline after PSM (severe: 1537 [4.7%] vs 1569 [4.8%]; standard mean difference, 0.001) (eTable 4 in Supplement 1).

**Table 4.** Comparison of Falsification Outcome Among Patients With Type 2 Diabetes and With or Without Preexisting Diabetic Retinopathy With vs Without GLP-1 RA Prescriptions

Outcome^a^	With GLP-1 RA prescriptions, No. (%) (n = 185 066)	Without GLP-1 RA prescriptions, No. (%) (n = 185 066)	RD (95% CI)	RRR, %	HR (95% CI)	*P* value	E-value for HR
Appendicitis in entire cohort	389 (0.2)	400 (0.2)	−0.000 (−0.000 to 0.000)	2.75	1.00 (0.87 to 1.15)	.99	1.03
Appendicitis in a subgroup with preexisting DR	108 (0.1)	112 (0.1)	−0.000 (−0.001 to 0.001)	3.57	0.98 (0.75 to 1.28)	.88	1.17

Abbreviations: DR, diabetic retinopathy; GLP-1 RA, glucagon-like peptide-1 receptor agonist; HR, hazard ratio; RD, risk difference; RRR, relative risk reduction.

^a^ After propensity matching.

## Discussion

To our knowledge, the findings of this study provide some of the first evidence that, among individuals with T2D, the use of GLP-1 RAs was associated with a lower risk of sight-threatening complications, including PDR, DME, and neovascular glaucoma, despite a marginally increased incidence of any DR. Among individuals with preexisting DR, GLP-1 RAs were not associated with progression to PDR and new-onset DME but associated with reduced incidence of vitreous hemorrhage and neovascular glaucoma. The risk of ischemic optic nerve disease, including NAION, was similar for individuals with and without GLP-1 RA prescriptions and with or without preexisting DR. We also found that those with GLP-1 RA prescriptions had a reduced need for medical, surgical, or laser-based interventions and a lower incidence of all-cause blindness regardless of preexisting DR.

Our clinical analysis revealed a modest increase in the incidence of DR but a lower risk of progression to sight-threatening disease in patients with preexisting DR. Prior studies of incretin mimetics have produced mixed results regarding their role in diabetic eye disease complications. No safety signals related to DR were reported in the EXSCEL (Exenatide Study of Cardiovascular Event Lowering) and the LEADER (Liraglutide Effect and Action in Diabetes: Evaluation of Cardiovascular Outcome Results) trials.^17,18^ In contrast, the SUSTAIN (Semaglutide Unabated Sustainability in Treatment of Type 2 Diabetes) series of phase 3 trials found a small increased risk of DR as well as the need for retinal laser treatment or intravitreal injections, vitreous hemorrhage, or diabetes-related blindness.^7^ Notably, the PIONEER-6 (A Trial Investigating the Cardiovascular Safety of Oral Semaglutide in Subjects With Type 2 Diabetes)^1^ and AWARD-11 (Efficacy and Safety of Dulaglutide 3.0 mg and 4.5 mg Versus Dulaglutide 1.5 mg in Metformin-Treated Patients With Type 2 Diabetes)^19^ studies did not show a worsening of DR or other retinal complications. A post hoc analysis of SUSTAIN-6 linked worsening of DR to the rate of glycemic improvement, with the semaglutide group experiencing HbA_1c_ reductions of 1.9% to 2.5% by week 16, compared with slower reductions in the placebo group.^20^ Additionally, a trial in patients with T2D and a body mass index (calculated as weight in kilograms divided by height in meters squared) of 27 reported worsening of DR in 4.0% of patients who were treated with semaglutide vs 2.7% of patients who received placebo.^21^ This difference was attributed to baseline DR history and insulin use. However, patients with uncontrolled or unstable DR were excluded from this trial. While our study balanced cohorts by insulin use and preexisting DR, we could not evaluate the rate of glycemic improvement or adjust for baseline DR stability or severity.

The potential protective properties of GLP-1 RAs against sight-threatening DR complications are likely multifactorial. While the association between HbA_1c_ and progression of DR may resemble that seen in type 1 diabetes after initiating intensive insulin treatment or in T2D with tight blood glucose control,^22,23^ the reduction of risk in sight-threatening DR complications also occurs even in the shorter term using these agents.^22,23^ GLP-1 RAs may exert direct and indirect benefits on diabetic complications, primarily through improved glycemic control, which is reflected by decreased HbA_1c_ levels. Secondary benefits of GLP-1 RAs include weight loss and prevention of cardiovascular and chronic kidney disease,^2,3,4,5,6^ which may provide additional vascular benefits in patients with diabetic eye disease. Experimental studies have shown that GLP-1 RAs can reverse early retinal changes associated with DR, restoring blood-retinal barrier integrity and preventing retinal cell death.^24,25,26^

Patients with diabetes are at an increased risk of NAION.^27^ A recent retrospective study raised concerns about a potential association between semaglutide use and NAION in the first year, suggesting that these agents might affect glucose metabolism or availability, risking damage to the highly metabolically active anterior optic nerve.^10^ However, a larger cohort study did not find any increased risk of NAION associated with GLP-1 RA use in patients with T2D, with or without obesity.^12^ We did not observe a statistically significant difference in the incidence of NAION between groups, likely due to the limited number of events and resulting imprecision in effect estimates.

In our study, although the use of GLP-1 RAs was associated with a modest increase in the rate of incident DR (0.2% increase) over 2 years, the absolute number of new DR diagnoses was low at 2.7%, likely below the expected annual incidence in US patients with T2D.^28^ Furthermore, GLP-1 RA use was associated with a 24.2% reduction in the risk of vision loss progressing to blindness, with 30.2% reduction in patients with preexisting DR. Diabetes is a leading cause of blindness in the US, and more advanced DR at diagnosis substantially increases the risk of blindness.^29,30,31^ Untreated PDR or DME also poses higher risks.^32,33^ Improving HbA_1c_ is the key factor in delaying DR progression and preventing sight-threatening complications.^23,34^ With advances in DR screening reducing visual impairment, our findings suggest that GLP-1 RAs could further contribute to these positive patterns.^35^

The cost of GLP-1 RAs must be weighed against the potential of these agents to lower the costs associated with the treatment of diabetes and its complications.^36^ Our study found that many interventions for DR, including intravitreal injections, panretinal laser photocoagulation, and vitrectomy for DR, were less frequently needed by patients with GLP-1 RA prescriptions compared with those without GLP-1 RA prescriptions. We also found a lower rate of initiation of treatment with anti-VEGF inhibitors, a class of agents commonly used to treat DR and DME that costs Medicare Part D nearly $1 billion per year.^37^ The compliance rate, considering the adverse effect profile of GLP-1 RAs, should be factored in when prescribing them. Studies that used administrative claims data from the US, UK, and Europe reported that approximately 20% to 50% of patients receiving GLP-1 RAs discontinued treatment within 12 months of initiation.^38^ Future studies should examine how compliance and adherence affect effectiveness end points to improve understanding of the clinical effectiveness outcomes of GLP-1 RAs, particularly considering gastrointestinal adverse effects. Our study adds to the growing body of evidence that GLP-1 RAs show promise in reducing the cost of treating diabetes.

### Limitations

Our study is limited by the anonymized data available from the electronic health record systems of participating health care organizations, which may have varying coding practices and data completeness, potentially affecting data quality. While the TriNetX database allows for the analysis of large T2D cohorts treated with GLP-1 RAs in clinical settings, it may not capture intraclass and interclass differences between medications and prescribing patterns. Although we used PSM to control for metabolic and medical factors, it is unclear if our findings apply to individuals with different metabolic phenotypes or to those using GLP-1 RAs for off-label indications. The study is also limited by the lack of longitudinal HbA_1c_ measurements at fixed time intervals that would allow assessment of glycemic changes over the follow-up period. As a result, we could not directly evaluate the potential implications of rapid glucose lowering for the observed outcomes, a mechanism hypothesized to contribute to the initial worsening of DR with GLP-1 RA use. Due to the aggregate nature of our data, we were unable to determine the precise number of individuals excluded due to inadequate follow-up. Our analysis did not capture data on the switching or discontinuation of GLP-1 RAs, which may affect treatment patterns and outcomes. Additionally, certain demographic groups, such as Asian, Black, and Hispanic individuals or patients with lower income, are less likely to receive GLP-1 RA prescriptions, while patients using these medications may have greater health care access or diabetes self-efficacy.^36,39^ We were unable to account for all factors in social determinants of health, including access to eye specialists, which could have changed our results. Our analysis also did not control for other retinal conditions unrelated to diabetes that may have required treatment. Lastly, the findings on the association between GLP-1 RA use and lower rates of new-onset blindness were based on administrative coding of visual impairment, not eye examination data. Future studies are needed to quantify both the extent and types of blindness that may be prevented through the use of GLP-1 RAs.

## Conclusions

This retrospective cohort study showed that while GLP-1 RA use was associated with a slight increase in incident DR, fewer patients experienced progression to sight-threatening stages of DR, developed DR complications, or required invasive treatments. These findings suggest that GLP-1 RAs may be a factor in reduced rate of vision loss leading to blindness, even among individuals with preexisting DR. It is crucial that all patients with T2D treated with GLP-1 RAs, regardless of preexisting DR, receive regular screening and monitoring for potential complications of T2D.

## Notes

Supplement 1. **eAppendix.** Cohort Definition
eMethods
**eTable 1.** Standardized Codes, and Data Types for Baseline Covariates Used in the TriNetX Database **eTable 2.** Outcome Definitions and Corresponding ICD/CPT Codes **eTable 3.** Comparison of Ophthalmologic Endpoints Among GLP-1 RA Users With <10 Years Versus >10 Years of Type 2 Diabetes **eTable 4.** Baseline Adjustment of Diabetic Retinopathy Severity Among GLP-1 RA Users and Non-users in the Pre-existing DR Subgroup

Supplement 2.
Data Sharing Statement
