# Supplementary material for: GLP-1 Receptor Agonists and Sight-Threatening Ophthalmic Complications in Patients With Type 2 Diabetes
Source: JAMA Netw Open. 2025 Aug 11;8(8):e2526321. doi: 10.1001/jamanetworkopen.2025.26321 (PMC12340654; doi:10.1001/jamanetworkopen.2025.26321)
Supplement: Supplement 1. — eAppendix. Cohort Definition eMethods eTable 1. Standardized Codes, and Data Types for Baseline Covariates Used in the TriNetX Database eTable 2. Outcome Definitions and Corresponding ICD/CPT Codes eTable 3. Comparison of Ophthalmologic Endpoints Among GLP-1 RA Users With <10 Years Versus >10 Years of Type 2 Diabetes eTable 4. Baseline Adjustment of Diabetic Retinopathy Severity Among GLP-1 RA Users and Non-users in the Pre-existing DR Subgroup [file jamanetwopen-e2526321-s001.pdf]

Supplemental Online Content

Ramsey DJ, Makwana B, Dani SS, et al. Glucagon-like peptide-1 receptor agonists and risk of sight-threatening ophthalmic complications in patients with type 2 diabetes. *JAMA Netw Open*. 2025;8(8):e2526321.
doi:10.1001/jamanetworkopen.2025.26321

eAppendix. Cohort Definition

eMethods

eTable 1. Standardized Codes, and Data Types for Baseline Covariates Used in the TriNetX Database

eTable 2. Outcome Definitions and Corresponding ICD/CPT Codes

eTable 3. Comparison of Ophthalmologic Endpoints Among GLP-1 RA Users With <10 Years Versus >10 Years of Type 2 Diabetes

eTable 4. Baseline Adjustment of Diabetic Retinopathy Severity Among GLP-1 RA Users and Non-users in the Pre-existing DR Subgroup

This supplemental material has been provided by the authors to give readers additional information about their work.

© 2025 Ramsey DJ et al. *JAMA Network Open*

eAppendix.

Cohort definition

This section lists all terms used in the definitions of the two cohorts.

Query Criteria for Cohort 1 (GLP-1 RA Cohort)

This query was run on the network Research with 94 HCO(s) queried and 93 HCO(s) responded. A total of 62 network providers responded with patients. The final cohort included 280,593 patients who matched the query criteria listed in the table below.

**Ungrouped terms**					
	must have		demographics	Age	Age (at least 18 years (most recent occurrence))
**Group 1**					
	**Group 1A**				
	must have		diagnosis	UMLS:ICD10CM:E11	Type 2 diabetes mellitus
		and any of	laboratory	TNX:9037	Hemoglobin A1c/Hemoglobin.total in Blood (at least 6.60 %)
			laboratory	UMLS:LNC:4548-4	Hemoglobin A1c/Hemoglobin.total in Blood (at least 6.60 %)
		and any of	medication	NLM:RXNORM:1991302	semaglutide
			medication	NLM:RXNORM:1440051	lixisenatide
			medication	NLM:RXNORM:2601723	tirzepatide
			medication	NLM:RXNORM:1551291	dulaglutide
			medication	NLM:RXNORM:475968	liraglutide
			medication	NLM:RXNORM:60548	exenatide
	date constraint		The terms in this group occurred between Jan 1, 2015 and Sep 30, 2022		
	event relationship		Any instance of Group 1B occurred at least 6 months after any instance of Group 1A		
	**Group 1B**				
	must have	any of	medication	NLM:RXNORM:1991302	semaglutide
			medication	NLM:RXNORM:1440051	lixisenatide
			medication	NLM:RXNORM:2601723	tirzepatide

		medication	NLM:RXNORM:1551291	dulaglutide
		medication	NLM:RXNORM:475968	liraglutide
		medication	NLM:RXNORM:60548	exenatide

Query Criteria for Cohort 2 (No GLP-1 RA cohort)

This query was run on the network Research with 94 HCO(s) queried and 94 HCO(s) responded. A total of 75 providers(s) responded with patients. The final cohort included 1,399,684 patients who matched the query criteria listed in the table below.

**Ungrouped terms**				
must have		demographics	Age	Age (at least 18 years (most recent occurrence))
cannot have		medication	NLM:RXNORM:475968	liraglutide
	or	medication	NLM:RXNORM:1440051	lixisenatide
	or	medication	NLM:RXNORM:2601723	tirzepatide
	or	medication	NLM:RXNORM:60548	exenatide
	or	medication	NLM:RXNORM:1991302	semaglutide
	or	medication	NLM:RXNORM:1551291	dulaglutide
**Group 1**				
**Group 1A**				
must have		diagnosis	UMLS:ICD10CM:E11	Type 2 diabetes mellitus
	and any of	laboratory	TNX:9037	Hemoglobin A1c/Hemoglobin.total in Blood (at least 6.60 % (most recent occurrence))
		laboratory	UMLS:LNC:4548-4	Hemoglobin A1c/Hemoglobin.total in Blood (at least 6.60 % (most recent occurrence))
date constraint		The terms in this group occurred between Jan 1, 2015 and Sep 30, 2022		

© 2025 Ramsey DJ et al. *JAMA Network Open*

eMethods

Propensity Score Matching: Entire Cohort

Propensity score matching was performed on 41 characteristic(s). In the Demographics category patients were matched on Age at Index, Female, Not Hispanic or Latino, Hispanic or Latino, Unknown Ethnicity, White, Black or African American, Unknown Race, Asian characteristic(s). In the Diagnosis category patients were matched on Disorders of lipoprotein metabolism and other lipidemias, Hypertensive diseases, Ischemic heart diseases, Atrial fibrillation and flutter, Cerebral infarction, Other peripheral vascular diseases, Atherosclerosis of native arteries of the extremities, Chronic kidney disease (CKD), Chronic lower respiratory diseases, Benign neoplasms, except benign neuroendocrine tumors, Neoplasms, Personal history of nicotine dependence, Tobacco use characteristic(s). In the Medication category patients were matched on INSULIN, metformin, glipizide, sitagliptin, empagliflozin, canagliflozin, glyburide, dapagliflozin, tirzepatide, ANTILIPEMIC AGENTS, LOOP DIURETICS, spironolactone, ACE INHIBITORS, ANGIOTENSIN II INHIBITOR, sacubitril, ANTINEOPLASTICS characteristic(s). In the Laboratory category patients were matched on Cholesterol in LDL [Mass/volume] in Serum or Plasma, Iron [Mass/volume] in Serum or Plasma, BMI characteristic(s). Characteristics of the cohorts before and after matching are summarized in the table below.

Patient count before and after propensity score matching

Cohort	Patient count before matching	Patient count after matching
1 - On GLP DM 2015-22	280,593	185,066
2 - No GLP DM 2015-22	1,399,684	185,066

Propensity score density function - Before and after matching (cohort 1 - purple, cohort 2 - green)

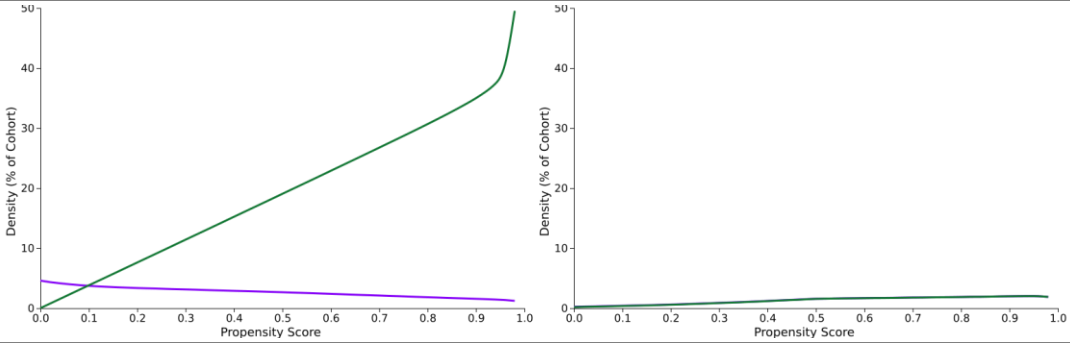

Propensity Score Matching: Prior DR subgroup

Propensity score matching was performed on 44 characteristic(s). In the Demographics category patients were matched on Age at Index, Female, Not Hispanic or Latino, Hispanic or Latino, Unknown Ethnicity, White, Black or African American, Unknown Race, Asian characteristic(s). In the Diagnosis category patients were matched on Disorders of lipoprotein metabolism and other lipidemias, Hypertensive diseases, Ischemic heart diseases, Atrial fibrillation and flutter, Cerebral infarction, Other peripheral vascular diseases, Atherosclerosis of native arteries of the extremities, Chronic kidney disease (CKD), Chronic lower respiratory diseases, Benign neoplasms, except benign neuroendocrine tumors, Neoplasms, Personal history of nicotine dependence, Tobacco use, Heart failure, Diastolic (congestive) heart failure, Systolic (congestive) heart failure characteristic(s). In the Medication category patients were matched on INSULIN, metformin, glipizide, sitagliptin, empagliflozin, canagliflozin, glyburide, dapagliflozin, tirzepatide, ANTILIPEMIC AGENTS, LOOP

© 2025 Ramsey DJ et al. *JAMA Network Open*

DIURETICS, spironolactone, ACE INHIBITORS, ANGIOTENSIN II INHIBITOR, sacubitril, ANTINEOPLASTICS characteristic(s). In the Laboratory category patients were matched on Cholesterol in LDL [Mass/volume] in Serum or Plasma, Iron [Mass/volume] in Serum or Plasma, BMI characteristic(s). Characteristics of the cohorts before and after matching are summarized in the table below.

Patient count before and after propensity score matching

Cohort	Patient count before matching	Patient count after matching
1 - Prior DR any on GLP	44,241	32,695
2 - Prior DR any NO GLP	124,760	32,695

Propensity score density function - Before and after matching (cohort 1 - purple, cohort 2 - green)

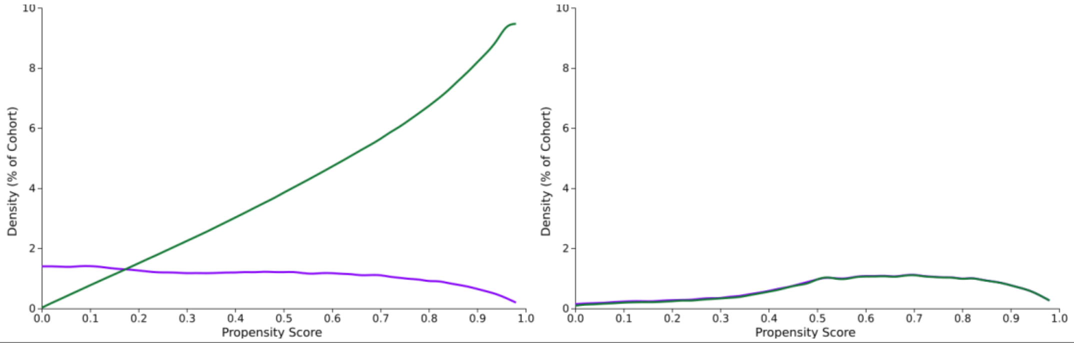

eTable 1. Standardized Codes, and Data Types for Baseline Covariates Used in the TriNetX Database

Covariate	Code	Data Type
**Demographics**		
Age, years	AI	Continuous
Female	F	Yes/No
White	2106-3	Yes/No
Non-Hispanic	2186-5	Yes/No
Hispanic or Latino	2135-2	Yes/No
Black or African American	2054-5	Yes/No
Asian	2028-9	Yes/No
**Comorbidities**		
Dyslipidemia	E78	Yes/No
Hypertension	I10-I1A	Yes/No
Ischemic Heart Diseases	I20-I25	Yes/No
Atrial fibrillation/flutter	I48	Yes/No
Ischemic stroke	I63	Yes/No
Peripheral Vascular Diseases	I70-I79	Yes/No
Chronic kidney disease	N18	Yes/No
Chronic Obstructive Pulmonary Diseases	J40-J4A	Yes/No
Benign Neoplasms	D10-D36	Yes/No
Malignant Neoplasms	C00-D49	Yes/No
Personal history of nicotine dependence	Z87.891	Yes/No
Tobacco use	Z72.0	Yes/No
**Medications**		
Insulin	HS501	Yes/No
Metformin	6809	Yes/No
Glipizide	4821	Yes/No
Sitagliptin	593411	Yes/No
Empagliflozin	1545653	Yes/No
Canagliflozin	1373458	Yes/No
Glyburide	4815	Yes/No
Dapagliflozin	1488564	Yes/No
Tirzepatide	2601723	Yes/No
Statins	CV350	Yes/No
Loop diuretics	CV702	Yes/No
Spironolactone	9997	Yes/No
ACE-inhibitors	CV800	Yes/No
Angiotensin II Receptor Inhibitors	CV805	Yes/No
Sacubitril	1656328	Yes/No
Antineoplastics	AN000	Yes/No
**Lab Values**		
LDL Cholesterol	9002	
BNP > 150 pg/ml	9003	Yes/No
NT-proBNP > 450 pg/ml	9072	Yes/No
Hemoglobin A1c ≥6.6%	9037	Yes/No
Iron (mcg/dL)	9043	Continuous
C-Reactive Protein (mg/L)	9063	Continuous
Left Ventricular Ejection Fraction (%)	2003	Continuous

BMI ≥ 30 kg/m^2	9083	Yes/No
**Abbreviations: ACE -** Angiotensin-converting enzyme; **LDL -** Low-density lipoprotein; **BNP -** Brain natriuretic peptide; **CRP -** C-Reactive protein; **BMI -** body mass index		

© 2025 Ramsey DJ et al. *JAMA Network Open*

eTable 2. Outcome Definitions and Corresponding ICD/CPT codes

Study Outcomes	ICD/CPT/RXNORM codes	Description
Diabetic Retinopathy (DR)	ICD10CM:E11.31	Type 2 diabetes mellitus with unspecified diabetic retinopathy
	ICD10CM:E11.32	Type 2 diabetes mellitus with mild nonproliferative diabetic retinopathy
	ICD10CM:E11.33	Type 2 diabetes mellitus with moderate nonproliferative diabetic retinopathy
	ICD10CM:E11.34	Type 2 diabetes mellitus with severe nonproliferative diabetic retinopathy
	ICD10CM:E11.35	Type 2 diabetes mellitus with proliferative diabetic retinopathy
	ICD10CM:E11.37	Type 2 diabetes mellitus with diabetic macular edema, resolved following treatment
NAION	ICD10CM:H47.01	Ischemic optic neuropathy
NAION (Broadly Defined)	ICD10CM:H47.01	Ischemic optic neuropathy
	ICD10CM:H47.10	Unspecified papilledema
	ICD10CM:H47.12	Papilledema associated with decreased ocular pressure
	ICD10CM:H47.13	Papilledema associated with retinal disorder
	ICD9CM:377.0	Papilledema
	ICD10CM:H46	Optic neuritis
Neovascular Glaucoma	ICD10CM:H40.53X1	Glaucoma secondary to other eye disorders, bilateral, mild stage
	ICD10CM:H40.53X2	Glaucoma secondary to other eye disorders, bilateral, moderate stage
	ICD10CM:H40.53X4	Glaucoma secondary to other eye disorders, bilateral, indeterminate stage
	ICD10CM:H40.53X3	Glaucoma secondary to other eye disorders, bilateral, severe stage
	ICD10CM:H40.53X0	Glaucoma secondary to other eye disorders, bilateral, stage unspecified
	CPT:66710	Ciliary body destruction; cyclophotocoagulation, transscleral
	CPT:66711	Ciliary body destruction; cyclophotocoagulation, endoscopic, without concomitant removal of crystalline lens
	CPT:67228	Treatment of extensive or progressive retinopathy (eg, diabetic retinopathy), photocoagulation
Blindness	ICD10CM:H54	Blindness and low vision
Severe NPDR	ICD10CM:E11.349	Type 2 diabetes mellitus with severe nonproliferative diabetic retinopathy without macular edema
	ICD10CM:E11.341	Type 2 diabetes mellitus with severe nonproliferative diabetic retinopathy with macular edema
Proliferative Diabetic Retinopathy	ICD10CM:E11.35	Type 2 diabetes mellitus with proliferative diabetic retinopathy
	ICD10CM:E11.311	Type 2 diabetes mellitus with unspecified diabetic retinopathy with macular edema
	ICD10CM:E11.321	Type 2 diabetes mellitus with mild nonproliferative diabetic retinopathy with macular edema
	ICD10CM:E11.331	Type 2 diabetes mellitus with moderate nonproliferative diabetic retinopathy with macular edema
	ICD10CM:E11.341	Type 2 diabetes mellitus with severe nonproliferative diabetic retinopathy with macular edema
	ICD10CM:E11.351	Type 2 diabetes mellitus with proliferative diabetic retinopathy with macular edema

© 2025 Ramsey DJ et al. *JAMA Network Open*

**DME**	UMLS:ICD10CM:E11.311	Type 2 diabetes mellitus with unspecified diabetic retinopathy with macular edema
	UMLS:ICD10CM:E11.321	Type 2 diabetes mellitus with mild nonproliferative diabetic retinopathy with macular edema
	UMLS:ICD10CM:E11.331	Type 2 diabetes mellitus with moderate nonproliferative diabetic retinopathy with macular edema
	UMLS:ICD10CM:E11.341	Type 2 diabetes mellitus with severe nonproliferative diabetic retinopathy with macular edema
	UMLS:ICD10CM:E11.351	Type 2 diabetes mellitus with proliferative diabetic retinopathy with macular edema
**Vitreous hemorrhage**	ICD10CM:H43.1	Vitreous hemorrhage
**Laser Photocoagulation**	UMLS:CPT:67228	Treatment of extensive or progressive retinopathy (eg, diabetic retinopathy), photocoagulation
**Vitrectomy**	CPT:67036	Vitrectomy, mechanical, pars plana approach
	CPT:67039	Vitrectomy, mechanical, pars plana approach; with focal endolaser photocoagulation
	CPT:67040	Vitrectomy, mechanical, pars plana approach; with endolaser panretinal photocoagulation
	CPT:67041	Vitrectomy, mechanical, pars plana approach; with removal of preretinal cellular membrane (eg, macular pucker)
	CPT:67042	Vitrectomy, mechanical, pars plana approach; with removal of internal limiting membrane of retina (eg, for repair of macular hole, diabetic macular edema), includes, if performed, intraocular tamponade (ie, air, gas or silicone oil)
	CPT:67113	Repair of complex retinal detachment (eg, proliferative vitreoretinopathy, stage C-1 or greater, diabetic traction retinal detachment, retinopathy of prematurity, retinal tear of greater than 90 degrees), with vitrectomy and membrane peeling, including, when performed, air, gas, or silicone oil tamponade, cryotherapy, endolaser photocoagulation, drainage of subretinal fluid, scleral buckling, and/or removal of lens
**Treatment with Anti-VEGF Medications**	RXNORM:253337	bevacizumab (Route: Ophthalmic Product)
	RXNORM:595060	ranibizumab (Route: Ophthalmic Product)
	RXNORM:1232150	aflibercept (Route: Ophthalmic Product)
	RXNORM:2204915	brolucizumab
	RXNORM:2591519	faricimab
**Composite Treatment Outcome**	UMLS:CPT:67028	Intravitreal injection of a pharmacologic agent (separate procedure)
	UMLS:CPT:67228	Treatment of extensive or progressive retinopathy (eg, diabetic retinopathy), photocoagulation
	UMLS:CPT:67036	Vitrectomy, mechanical, pars plana approach
	UMLS:CPT:67039	Vitrectomy, mechanical, pars plana approach; with focal endolaser photocoagulation
	UMLS:CPT:67040	Vitrectomy, mechanical, pars plana approach; with endolaser panretinal photocoagulation
	UMLS:CPT:67041	Vitrectomy, mechanical, pars plana approach; with removal of preretinal cellular membrane (eg, macular pucker)

© 2025 Ramsey DJ et al. *JAMA Network Open*

	UMLS:CPT:67042	Vitrectomy, mechanical, pars plana approach; with removal of internal limiting membrane of retina (eg, for repair of macular hole, diabetic macular edema), includes, if performed, intraocular tamponade (ie, air, gas or silicone oil)
	UMLS:CPT:67113	Repair of complex retinal detachment (eg, proliferative vitreoretinopathy, stage C-1 or greater, diabetic traction retinal detachment, retinopathy of prematurity, retinal tear of greater than 90 degrees), with vitrectomy and membrane peeling, including, when performed, air, gas, or silicone oil tamponade, cryotherapy, endolaser photocoagulation, drainage of subretinal fluid, scleral buckling, and/or removal of lens
**Appendicitis**	ICD10CM:K35-K38	Diseases of appendix

© 2025 Ramsey DJ et al. *JAMA Network Open*

eTable 3. Comparison of Ophthalmologic Endpoints Among GLP-1 RA Users With < 10 Years Versus > 10 Years of Type 2 Diabetes

Outcomes	T2D Duration < 10 years (n = 68,424)*	T2D Duration > 10 years (n = 68,424)*	Hazard ratio (95% CI)	*p*-value
**DR**	2,458	2,492	0.985 (0.931, 1.041)	0.586
**PDR**	676	673	1.006 (0.904, 1.119)	0.917
**DME**	5,127	5,185	0.989 (0.952, 1.028)	0.589
**Neovascular Glaucoma**	349	349	1.001 (0.863, 1.162)	0.984
**NAION**	38	38	1.002 (0.639, 1.570)	0.994
**Vitreous hemorrhage**	795	802	0.992 (0.900, 1.095)	0.879
**Laser Photocoagulation**	534	539	0.992 (0.880, 1.118)	0.897
**Vitrectomy**	749	755	0.993 (0.898, 1.099)	0.898
**Treatment with Anti-VEGF Medications**	206	207	0.996 (0.822, 1.208)	0.971
**Composite Treatment Outcome^**	1,880	1,892	0.995 (0.933, 1.060)	0.868
**Blindness**	1,415	1,428	0.992 (0.922, 1.068)	0.833

***After propensity matching. Abbreviation:** **GLP-1 RA** - Glucagon-like Peptide-1 Receptor Agonist; **DR** - Diabetic Retinopathy; **NAION** - Non-Arteritic Anterior Ischemic Optic Neuropathy; **PDR** - Proliferative Diabetic Retinopathy; **VEGF** - Vascular Endothelial Growth Factor; **DME -** Diabetic Macular Edema.
^ **Composite treatment outcome** consists of treatment of diabetic retinopathy with anti-VEGF agents, panretinal laser photocoagulation, or vitrectomy for DR.

© 2025 Ramsey DJ et al. *JAMA Network Open*

eTable 4. Baseline Adjustment of Diabetic Retinopathy Severity Among GLP-1 RA Users and Non-Users in the Pre-Existing DR Subgroup

Diabetic Retinopathy Severity	On GLP-1 RAs (%; n = 32,695)	Not on GLP-1 RAs (%; n = 32,695)	Standardized Mean Difference
Unspecified DR	18,309 (56.0)	18,146 (55.5)	0.010
Mild nonproliferative DR	12,947 (39.6)	13,111 (40.1)	0.011
Moderate nonproliferative DR	4,087 (12.5)	4,185 (12.8)	0.010
Severe nonproliferative DR	1,537 (4.7)	1,569 (4.8)	0.001
PDR	5,133 (15.7)	5,100 (15.6)	0.003
DME	131 (0.4)	131 (0.4)	0.001

GLP-1 RA - Glucagon-like Peptide-1 Receptor Agonist; **DR** - Diabetic Retinopathy; **PDR** - Proliferative Diabetic Retinopathy; **DME -** Diabetic Macular Edema.

© 2025 Ramsey DJ et al. *JAMA Network Open*
