# Supplementary material for: GLP-1 Receptor Agonists and Sight-Threatening Ophthalmic Complications in Patients With Type 2 Diabetes
Source: JAMA Netw Open. 2025 Aug 11;8(8):e2526321. doi: 10.1001/jamanetworkopen.2025.26321 (PMC12340654; doi:10.1001/jamanetworkopen.2025.26321)
Supplement: Supplement 2. — Data Sharing Statement [file jamanetwopen-e2526321-s002.pdf]

Data Sharing Statement

Ramsey. Glucagon-Like Peptide-1 Receptor Agonists and Risk of Sight-Threatening Ophthalmic Complications in Patients With Type 2 Diabetes. *JAMA Netw Open*. Published August 11, 2025. doi:10.1001/jamanetworkopen.2025.26321

Data
Data available: No
